# A local drug delivery system prolongs graft survival by dampening T cell infiltration and neutrophil extracellular trap formation in vascularized composite allografts

**DOI:** 10.3389/fimmu.2024.1387945

**Published:** 2024-06-03

**Authors:** Isabel Arenas Hoyos, Anja Helmer, Anaïs Yerly, Ioana Lese, Stefanie Hirsiger, Lei Zhang, Daniela Casoni, Luisana Garcia, MariaFrancesca Petrucci, Sabine E. Hammer, Tereza Duckova, Yara Banz, Matteo Montani, Mihai Constantinescu, Esther Vögelin, Gregor Bordon, Simone Aleandri, Jean-Christophe Prost, Adriano Taddeo, Paola Luciani, Robert Rieben, Nicoletta Sorvillo, Radu Olariu

**Affiliations:** ^1^ Department for BioMedical Research, University of Bern, Bern, Switzerland; ^2^ Department of Plastic and Hand Surgery, Inselspital, Bern University Hospital, Bern, Switzerland; ^3^ Institute of Immunology, University of Veterinary Medicine Vienna, City Bern, Austria; ^4^ Institute of Pathology, Inselspital, Bern University Hospital, Bern, Switzerland; ^5^ Department of Chemistry, Biochemistry and Pharmaceutical Sciences, University of Bern, Bern, Switzerland; ^6^ University Institute of Clinical Chemistry, Inselspital, Bern University Hospital, University of Bern, Vienna, Switzerland

**Keywords:** vascularized composite allotransplantation (VCA), transplantation immunology, tacrolimus, local immunosuppression, calcineurin inhibitors (CNIs), drug delivery systems (DDSs), porcine model, neutrophil extracellular traps (NETs)

## Abstract

**Introduction:**

The standard treatment for preventing rejection in vascularized composite allotransplantation (VCA) currently relies on systemic immunosuppression, which exposes the host to well-known side effects. Locally administered immunosuppression strategies have shown promising results to bypass this hurdle. Nevertheless, their progress has been slow, partially attributed to a limited understanding of the essential mechanisms underlying graft rejection. Recent discoveries highlight the crucial involvement of innate immune components, such as neutrophil extracellular traps (NETs), in organ transplantation. Here we aimed to prolong graft survival through a tacrolimus-based drug delivery system and to understand the role of NETs in VCA graft rejection.

**Methods:**

To prevent off-target toxicity and promote graft survival, we tested a locally administered tacrolimus-loaded on-demand drug delivery system (TGMS-TAC) in a multiple MHC-mismatched porcine VCA model. Off-target toxicity was assessed in tissue and blood. Graft rejection was evaluated macroscopically while the complement system, T cells, neutrophils and NETs were analyzed in graft tissues by immunofluorescence and/or western blot. Plasmatic levels of inflammatory cytokines were measured using a Luminex magnetic-bead porcine panel, and NETs were measured in plasma and tissue using DNA-MPO ELISA. Lastly, to evaluate the effect of tacrolimus on NET formation, NETs were induced *in-vitro* in porcine and human peripheral neutrophils following incubation with tacrolimus.

**Results:**

Repeated intra-graft administrations of TGMS-TAC minimized systemic toxicity and prolonged graft survival. Nevertheless, signs of rejection were observed at endpoint. Systemically, there were no increases in cytokine levels, complement anaphylatoxins, T-cell subpopulations, or neutrophils during rejection. Yet, tissue analysis showed local infiltration of T cells and neutrophils, together with neutrophil extracellular traps (NETs) in rejected grafts. Interestingly, intra-graft administration of tacrolimus contributed to a reduction in both T-cellular infiltration and NETs. In fact, *in-vitro* NETosis assessment showed a 62–84% reduction in NETs after stimulated neutrophils were treated with tacrolimus.

**Conclusion:**

Our data indicate that the proposed local delivery of immunosuppression avoids off-target toxicity while prolonging graft survival in a multiple MHC-mismatch VCA model. Furthermore, NETs are found to play a role in graft rejection and could therefore be a potential innovative therapeutic target.

## 1 Introduction

Vascularized composite allotransplantation (VCA), such as hand and face grafts, refers to the transplantation of functional units containing multiple tissue types like skin, muscle, bone, and nerves. VCA offers unique restoration of both esthetics and functionality, which is not attainable with any other currently available reconstructive methods. After the first described hand transplantation attempt in 1964 (1), it took 34 years for VCA to become a clinical reality thanks to the advent of potent immunosuppressive therapies like calcineurin inhibitors (CNI) (2). Since then, the field has expanded greatly, with over 170 VCAs performed worldwide, including face, hand, uterus, and abdominal wall transplantations (3–6).

Optimal immunosuppression is the key to maintaining VCA graft survival. Currently, systemic administration of two or more immunosuppressive drugs, including CNIs such as tacrolimus (TAC), in combination with perioperative induction therapy is needed to prevent graft rejection (7). Due to their systemic distribution, these medications can cause a wide range of well-known side effects such as kidney toxicity and opportunistic infections (8, 9). Immunosuppression via targeted drug delivery systems presents a paradigm shift in managing immune-related disorders, offering distinct advantages over systemic immunosuppression (10, 11). A notable benefit lies in the precision of localized drug delivery, enabling therapeutic agents to directly target affected tissues or organs while minimizing systemic exposure. Different drug delivery systems (DDS) have been developed as carriers of immunosuppression therapy in VCA, ranging from hydrogels (12–16), to spheres/nanoparticles (17–20), *in-situ* forming implants (ISFI) (21) and disks (22–24). In contrast to other biomaterials, hydrogels have been widely used as drug delivery platforms as they offer the capacity to encapsulate substantial quantities of hydrophobic drugs like tacrolimus, with high biocompatibility, self-assembly and sustain drug release properties, among others, after local administration (25, 26). Our group developed a tacrolimus-based hydrogel drug delivery system as an alternative to conventional systemic TAC administration. This DDS, consisting of a triglycerol monostearate (TGMS) hydrogel loaded with tacrolimus (TGMS-TAC), is injected directly into the graft. Local release of tacrolimus from the hydrogel is mediated by esterases, matrix metalloproteinases (MMPs), and other proteases found in the extracellular space during inflammation. Compared to systemic tacrolimus treatment, intra-graft TGMS-TAC injections prolonged graft survival and reduced systemic off-target toxicity in a rodent model of VCA (12, 13, 27). A tacrolimus-based hydrogel has also been proven successful in a porcine VCA model (14). However, a single major histocompatibility complex (MHC)-mismatch was used, limiting its translational value for application in humans. In solid organ transplantation (SOT), MHC matching between donor and recipients is a common practice, since it has shown a pivotal role in determining the long-term outcome of the allografts (28). In a clinical VCA setting, contrary to SOT, a genetic match based on MHC compatibility between donors and recipients is unrealistic, as many other matching criteria (e.g., gender, age, and skin color) are also required. Thus, human VCAs are commonly performed with greater MHC disparities, with an average of 3–4/6 HLA-A, B, and DR loci mismatches (29), which are known to impact graft rejection (30). We therefore decided to assess TGMS-TAC in a multiple MHC-mismatched porcine VCA model that better resembles clinical reality.

Tacrolimus, a CNI targeting primarily T cells (31), is considered the backbone of most VCA maintenance regimens (32). This is especially relevant given that acute T cell-mediated rejection has been described to be predominant for both hand and face allotransplants (33, 34). Interestingly, although surgical and immunosuppressive protocols for VCA have been improved, vasculopathy and rejections still occur; with a reported incidence of up to 92% for acute rejection episodes within the first year (9, 35).

While adaptive immune cells (mainly T and B cells) have dominated transplant studies for years, recent findings have also identified important roles for innate immune cells (e.g. neutrophils) in organ transplantation (36). Neutrophils are the first cells to migrate to the transplanted organ upon rejection where their presence causes not only direct tissue injury but also triggers alloimmune responses through cross-talk with other graft-resident leukocytes, including T cells (37). During inflammation, neutrophils can release extracellular traps, weblike structures of decondensed DNA linked to cytoplasmic and granular proteins such as myeloperoxidase (MPO) and neutrophil elastase. These neutrophil extracellular traps (NETs) are known to participate in many inflammatory disorders from thrombosis to autoimmunity and cancer (38). Although NETs have recently been associated with ischemia-reperfusion injury and have been shown to mediate rejection in SOT (39–42), their role in VCA is unknown. Here, we investigated the effect of intra-graft administration of TGMS-TAC in a clinically relevant, multiple MHC-mismatch, large animal model of VCA, and evaluated the role of T cells, neutrophils, and NETs in graft rejection. We provide pre-clinical data on the feasibility and validity of this novel immunosuppressive approach in avoiding off-target toxicity and identify novel therapeutic targets that may prolong graft survival.

## 2 Materials and methods

### 2.1 Swine lymphocyte antigen typing

Donor and recipient pigs used in this study were genotyped for their swine lymphocyte antigen (SLA) class I and II haplotypes by running low-resolution PCR screening assays on ear punch biopsies (sows) and testicular tissues (boars). Genomic DNA was isolated using commercial kits following the manufacturer’s instructions (QIAamp DNA Micro Kit, Qiagen, Hilden, Germany; E.Z.N.A. Tissue DNA Kit, Omega Bio-tek, Inc., Norcross, GA, USA). SLA class I (SLA-1, SLA-2, and SLA-3) and SLA class II (DRB1, DQB1 and DQA) low-resolution haplotypes were identified by a sequence-specific primed PCR-based typing assay. The criteria and nomenclature used for SLA class I and SLA class II haplotyping were based on those proposed by the international SLA ISAG/IUIS-VIC nomenclature Committee in the IPD-MHC database of suids (www.ebi.ac.uk/ipd/mhc/group/SLA) (43, 44).

### 2.2 Preparation of TGMS-TAC, endotoxin test, and enzymatic drug release

TGMS-TAC was prepared as described previously (12). Briefly TGMS (AK Scientific, Union City CA, USA) and TAC (R&S Pharmchem Co., Shanghai, China) were dissolved in dimethyl sulfoxide (DMSO; Merck, Darmstadt, Germany). After complete dissolution, preheated MilliQ water (ThermoFisher Scientific, Waltham, MA, USA) was added to obtain the final formulation with a TAC concentration of 7 mg/mL and 20% v/v DMSO. TGMS TAC was subsequently transferred into Fisherbrand Luer-Lock sterile syringes (ThermoFisher). The formulation was tested for the presence of endotoxins using a Pierce Chromogenic Endotoxin Quant Kit (ThermoFisher). TGMS-TAC was prepared using endotoxin-free water and then tested according to the manufacturer’s instructions using 405 nm absorbance measured in a microplate reader (Tecan, Männedorf, Switzerland). TGMS-TAC enzyme-responsive drug release was measured as previously described. Briefly, 200 mL of TGMS-TAC hydrogel (TAC7 mg/mL) were diluted 1:4 in PBS. The dialysis bag (Float-A-Lyzer G2, Spectropore, molecular weight cutoff 8–10 kDa) was placed into a Falcon tube filled with PBS and pre-heated to 37°C. The tubes were kept at 37°C under horizontal shaking (50 rpm) for the whole duration of the release study. At different time points, aliquots were withdrawn for further analysis and the complete release medium replaced with fresh PBS. As an enzymatic challenge, on day 28 a volume of 100 mL of lipase type II solution (258 mg/mL) from porcine pancreas (SIGMA, Ref: SLCD5418, activity 388 units/mg) was added to these diluted samples and, at different time points, the TAC concentration was analyzed by HPLC.

### 2.3 Heterotopic hind limb transplantation and monitoring

All experimental procedures were approved by the Veterinary Office of the Canton of Bern (approval number: BE48/19), and animals were treated according to the Animal Welfare Act and Ordinance of the Swiss Animal Welfare Legislation during the entire experiment. Experimental protocols were refined according to 3R principles. Males and females were included in both donor and recipient groups. Animal well-being was regularly assessed by both scientists and board-certified veterinarians using a pre-designed score sheet for health and behavior appraisal to minimize any potential suffering and a clear guideline of early termination criteria.

**Figure 1:**
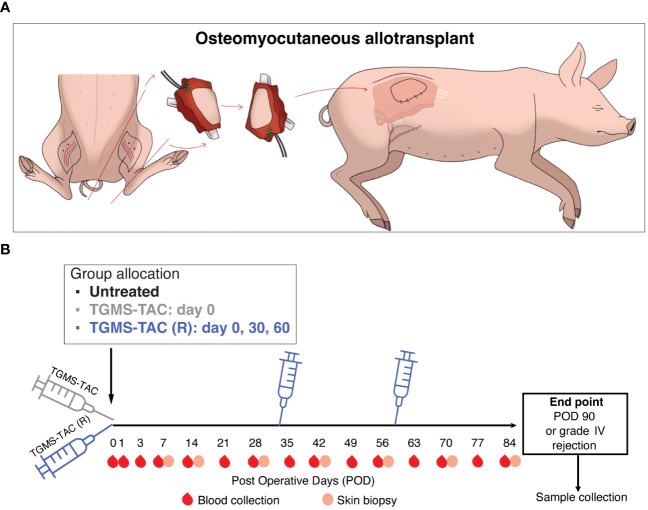
Experimental design. **(A)** Schematic representation of the modified heterotopic hind limb allotransplantation model, where an osteomyocutaneous flap containing graft-draining lymph nodes was transplanted into the flank of a recipient pig. **(B)** Recipient timeline. After transplantation, pigs were allocated to 3 groups: Group 1, untreated, with no immunosuppression. Group 2, subcutaneous intra-graft injection of TGMS-TAC, 20 mL/kg graft weight, only at POD 0 immediately after transplantation. Group 3, TGMS-TAC intra-graft injection, same dosing as in group 2, repeated injections at POD 0, 30 and 60. During follow-up, blood and tissue samples were collected at defined time points.

MHC-mismatched Swiss landrace pigs, aged 11–14 weeks, underwent a modified osteomyocutaneous flap allotransplant as a representative model of VCA (45). Briefly, a graft weighing approximately 800 grams, comprised of vascularized skin, muscles, knee joint, distal femur, and proximal tibia, was placed in the flank of a recipient pig, where the skin island of about 12x8 cm was exteriorized for graft monitoring purposes (**Figure 1A**). Before transplantation, the graft was weighed to determine the necessary dose of immunosuppressive medication. As previously described (46), a *port-a-cath* (Braun, Melsungen, Germany) central venous catheter was positioned during the surgical procedure in the external jugular vein with the port placed subcutaneously in the posterior neck region, in order to facilitate postoperative venous blood sampling without the need for sedating the animal for manipulation. Subsequently, pigs were allocated into three groups: group 1 (untreated), where no immunosuppressive therapy was given; group 2 (TGMS-TAC), where TGMS-TAC was given at a dose of 140 mg per kg of graft weight only at postoperative day (POD) 0 immediately after the procedure; and group 3 (TGMS-TAC (R)), where TGMS-TAC was injected at the same dose as in group 2 at PODs 0, 30 and 60. Evenly distributed 0.5 mL depots of TGMS-TAC were injected subcutaneously into the skin of the graft. All animals were followed up until either grade III rejection or POD 90 was reached. A 30-day dosing interval, as well as TGMS-TAC dose per kg of graft weight, was estimated based on the findings of tacrolimus tissue concentration after intra-graft TGMS-TAC therapy associated with graft rejection changes from prior published rat and porcine hind limb studies (13, 14). After transplantation, tissue and blood samples were collected at defined time points (**Figure 1B**).

### 2.4 Clinical and histopathological assessment of rejection

Macroscopic changes characteristics of graft rejection were assessed in the exteriorized skin of the VCA graft by two blinded investigators and were graded as previously described as 0 = no difference between graft skin and native skin, I = mild erythema, II = moderate erythema with the beginning of scaling and scabbing, III = severe erythema and scabbing with areas of epidermolysis, IV = full-thickness graft epidermolysis with areas of necrosis (47).

For histopathological analyses, skin and muscle samples collected from the graft at endpoint were fixed in 4% paraformaldehyde (PFA), sectioned, and stained with Hematoxylin and Eosin (H&E). Images were acquired using a Pannoramic 250 Flash III microscope (3D Histech, Budapest, Hungary) with the 10x objective. Skin rejection was assessed and classified using the Banff 2007 scoring for skin-containing composite tissue allografts (48). Muscle samples were analyzed for the presence of necrosis and/or atrophy and inflammatory infiltrates. A four-tiered system was used depicting the severity of changes from none (0), minimal (1) to moderate (2), and finally to extensive (3).

### 2.5 Tacrolimus levels

TAC levels were measured systemically (from blood) at baseline, POD1, 3, 7, and then weekly until endpoint. TAC levels in tissue were measured in skin biopsies collected from both the graft and contralateral skin at baseline, POD7, and then every 2 weeks until endpoint.

For systemic TAC levels, peripheral blood was collected from the vascular access port in EDTA-coated tubes (Sarstedt, Nümbrecht, Germany) and immediately processed. TAC concentrations in blood were assessed using the MS1100 Kit (ClinMass Complete Kit for Immunosuppressants in Whole Blood, RECIPE Chemicals + Instruments GmbH, Munich, Germany) and quantified by Ultra-High Performance Liquid Chromatography Tandem Mass Spectrometry (LC-MS/MS).

Skin biopsies, 5 mm size, from the graft and contralateral side were excised, weighed, snap-frozen, and stored at -80°C until further use. Care was taken to ensure that each skin sample was taken from a different region of the skin island over time. Local concentrations of TAC in the graft and contralateral side were measured from skin biopsies. Samples were processed and analyzed by LC-MS/MS as previously described (13, 14), where a modification in the calibration curve range was set to 30ng/mL, and the capillary and the cone voltages were set at 3.2 kV and 40 V, respectively.

### 2.6 Analyses of immunosuppression-related toxicity

To measure off-target toxicity, markers of liver and kidney function were analyzed from whole blood collected at baseline and then weekly until endpoint. Analyzed markers included cholesterol, triglyceride, creatinine, alanine aminotransferase, and aspartate aminotransferase. Paraffin-embedded kidney and liver samples retrieved from the animals at endpoint were sectioned and H&E stained for histopathological assessment of TAC-related changes by a blinded pathologist and compared to naive, age-matched landrace pigs. H&E slides were scanned using a Pannoramic 250 Flash III microscope (3D Histech, Budapest, Hungary) with the 10x objective.

### 2.7 Complete blood count, PBMC isolation, and flow cytometric analysis

A complete blood count was performed using EDTA-blood and an ADVIA 2120i analyzer (Siemens Healthcare AG, Zurich, Switzerland) running multispecies software (version 6.3.2-MS).

Peripheral blood from the vascular access port was collected in EDTA tubes and promptly centrifuged for plasma storage and peripheral blood mononuclear cells (PBMCs) collection. PBMCs were isolated using a sterile density gradient medium (Ficoll-Paque PLUS, MERCK, REF: 17144002) and stored at -150°C until use.

Circulating T cells were quantified from the previously isolated PBMCs through flow cytometry. Briefly, after washing with FACS buffer (PBS + 2% FBS) cells were incubated for 30 minutes with LIVE/DEAD Fixable Yellow Dead Cell Stain Kit 405nm. (ThermoFisher, Ref: L34959). Next, non-specific binding was blocked using FcR Blocking Reagent (MACS Ref 130–059-901). Subsequently, cells were incubated for 30 minutes with the following labeled antibodies: CD3-FITC (Clone PPT3. Bio-Rad, Ref: MCA5951F), CD3-RPE (Clone PPT3. Bio-Rad, Ref: MCA5951PE), CD4-PE Cy5 (Clone 74–12-4, ThermoFisher, Ref: MA5–28734), CD8b – FITC (Clone PPT23. Bio-Rad, Ref: MCA5954F). For intranuclear staining, cells were fixed, permeabilized, and washed using a FoxP3/Transcription Factor Staining Buffer set (eBioscience, Ref: 00–5523-00) and incubated 30 min with an anti-FoxP3 eFluor 450 antibody (Clone FJK-16s. ThermoFisher, Ref: 48–5773-82). Cells were acquired using a CytoFLEX S cytometer equipped with CytExpert Software (Beckman Coulter Life Science, California, United States). Data were analyzed using Flow-Jo software (Tri-Star, Ashland, United States).

### 2.8 Cytokine and complement measurement

Plasma levels of C3a were measured by ELISA using a porcine C3a ELISA kit (MyBioSource, Ref MBS2509360) according to the manufacturer’s instructions. Absorbance values were measured at 450 nm using a Varioskan LUX multimode microplate reader (ThermoFisher).

Cytokine levels were measured from EDTA plasma and skin samples collected at different time points. For tissue protein extraction, skin samples were weighed and manually homogenized in RIPA lysis and extraction Buffer (ThermoFisher Scientific, Ref: 89900) supplemented with protease inhibitors (Halt, ThermoFisher, Ref: 1861280). Samples were further lysed using a TissueLyser II (Qiagen), 2 cycles of 30 seconds at 20 Hz. After centrifugation, the supernatant was collected and stored at -20°C until used. A Luminex multiplex assay (MILLIPLEX Porcine Cytokine/Chemokine Magnetic Bead Panel. MERCK, Ref: PCYTMG-23K-13PX) was performed on tissue lysates and plasma collected at baseline and endpoint for the following markers: IFN-γ, IL-1α, IL-1β, IL-1ra, IL-2, IL-4, IL-6, IL-8, IL-10, IL-12, IL-18, and TNF-α.

### 2.9 Immunofluorescence staining

Skin and muscle samples collected at endpoint were embedded in TissueTec - O.C.T., (Sakura Finetek, Alphen aan den Rijn, The Netherlands) and cryostat sectioned at 5 µm. Tissue slides were stained with DAPI (4’,6-diamidino-2-phenylindole. Invitrogen, Ref: D1306) and the following antibodies: CD31 (Clone 377537. R&D system, Ref: MAB33871), CD3 (Clone PPT3. Biotech, Ref: 4510–13), C3b/c - FITC (Agilent Dako – Ref: F0201), Myeloperoxidase (Clone 2C7. Abcam, Ref: ab25989), H4Cit (Millipore, Ref: 07–596). The following secondary antibodies were used: goat anti-rat Alexa Fluor 680 (Invitrogen, Ref: A21096), donkey anti-rabbit Alexa Fluor 488 (Invitrogen, Ref: A32790), donkey anti-mouse Alexa Fluor 568 (Invitrogen, Ref: A10037). Slides were imaged using confocal microscopy (20x objective, Zeiss LSM 980, Zen Blue software), and processed using ImageJ software (https://imagej.nih.gov/ij/). For quantification of infiltrating T cells in tissue, the percentage of CD3+ cells was calculated as [number of CD3+ cells/total number of cells per field] x 100, from 5 representative immunofluorescence images/sample/pig.

### 2.10 Western blot

Neutrophils in allografts were assessed by western-blot analysis. Graft skin protein extractions were performed as described above and quantified using the Pierce Coomassie (Bradford) Protein Assay Kit (ThermoFisher, Ref: 23200). Equal amounts of protein (50 µg) were loaded and electrophoretically resolved using Bolt 4–12%, Bis-Tris gels (Invitrogen, Ref: NW04120BOX). Proteins were then transferred to PVDF membranes (Invitrogen, Ref: IB24001). Membranes were blocked using Intercept Blocking buffer (LI-COR, Ref: 927–70001), and then incubated with a primary antibody against MPO (Clone 2C7, Abcam, Ref: ab25989) followed by a goat anti-mouse, IRDye 800cW secondary antibody (LI-COR, Ref: 926–32210). Blots were imaged using the Odyssey Imaging System 9120 (LI-COR Biosciences, Nebraska, United States). An antibody against beta-actin (LI-COR, Ref: 926–42212) was used to assess sample loading. Bands were quantified using ImageJ software (https://imagej.nih.gov/ij/) by dividing the mean intensity value of the band of interest by the intensity of the loading control. The amount of MPO in allograft skin samples was calculated as a fold-change compared to the amount found in healthy naive tissue.

### 2.11 DNA-MPO ELISA

DNA-MPO complexes were measured by ELISA (49, 50) in EDTA-plasma and in graft skin lysate obtained as previously described. In brief, Nunc MaxiSorp plates were coated with 5 mg/mL of anti-myeloperoxidase (MPO) antibody (Clone 4A4. Bio-Rad, Ref: 0400–0002). Prior to sample addition, wells were blocked with 5% BSA for 2 hours. To increase assay sensitivity, sample NETs were digested using 2 U/mL of DNAse I (Sigma, DN25) for 15 minutes, after which 2.5 mM of EDTA was added to stop the digestion. Samples were incubated with a peroxidase-conjugated anti-DNA antibody from an ELISA kit for cell death detection (Roche, Ref: 11544675001) for 2 hours. After washing, TMB substrate (3,3’,5,5’-tetramethylbenzidine. ThermoFisher, Ref: N301) was added to each well and incubated for a maximum of 20 minutes. The reaction was stopped using 0.5M sulfuric acid. Complexes were quantified as optical density values at 405 nm using a Varioskan LUX multimode microplate reader. DNA-MPO levels were calculated as fold-change with respect to levels measured in pooled plasma or skin lysates obtained from 8 naive healthy pigs.

### 2.12 NET formation assay

Porcine and human neutrophils were both isolated from EDTA-blood from healthy individuals by density centrifugation with Histopaque-1119 (Merck, Ref: 11191) and density-gradient centrifugation with Percoll (Merck, Ref: P4937). The purity of isolated neutrophils was confirmed using the Sysmex KX21N Hematology Analyzer (Sysmex Corporation. Hyogo, Japan). To assess the effect of TAC on NET formation, neutrophils were cultured in RPMI 1640 medium (Gibco, Ref: 72400047) and stimulated with 5 µM ionomycin (ThermoFisher, Ref: I24222) in the presence of different concentrations (0 - 100 ng/mL) of TAC (R&S Pharmchem, Ref: 104987–11-3). To identify NETs, cellular DNA was stained with DAPI and then visualized using confocal microscopy (Zeiss LSM 980). NETs were defined morphologically as neutrophils with extruded DNA content in a web-like structure and quantified as percentages using [number of NETs + neutrophils/total neutrophils per field] x 100, from 5 representative images/sample.

### 2.13 Statistical analysis

Inter-group t-tests and analyses of variance (ANOVA) were performed using Prism software (GraphPad, La Jolla, CA, United States). The following symbols represent statistical significance based on *p* values determined by the specific group tests described in each figure legend: *p<0.05; **p<0.01; ***p<0.001; and ****p<0.0001.

## 3 Results

### 3.1 Repeated intra-graft TGMS-TAC administrations prolong graft survival in a porcine VCA model with multiple MHC mismatches

To evaluate the effect of TGMS-TAC on VCA in a clinically relevant setting, a series of heterotopic limb allotransplantations were performed in Swiss landrace pigs (**Figure 1A**). Donor and recipient pigs were selected based on their Swine Leukocyte Antigen (SLA) typing to achieve a constant mismatch of 8–10 out of 12 alleles within and between groups (**Supplementary Figure S1**). TGMS-TAC is an enzyme-responsive hydrogel, where TAC release is boosted by the presence of proteolytic enzymes such as matrix metalloproteinases (MMPs) and lipase, which are upregulated in tissue during inflammation (**Supplementary Figure S2A**). We tested the inflammation-based delivery profile of TGMS-TAC and, as shown in **Supplementary Figure S2B**, found a large cumulative increase in TAC release upon the addition of lipase. Next, to assess the *in-vivo* stability of the hydrogel after intra-graft injection, a syngeneic transplant (n = 1, MHC-mismatch 0/12), where no immunological challenge is expected, was performed. After transplantation, the recipient animal received a single dose of TGMS-TAC and was then followed until either postoperative day (POD) 90 or grade III rejection. As expected, the syngeneic transplanted animal reached 90 days of rejection-free graft survival. Ultrasound evaluation of the graft 30 days after injection of the hydrogel confirmed the presence of multiple intra-graft depots of TGMS-TAC (**Supplementary Figure S2C**), indicating that without an inflammatory environment, the hydrogel is stable, and TAC is not significantly released. In addition, no TAC levels were observed in circulation (data not shown).

**Figure 2:**
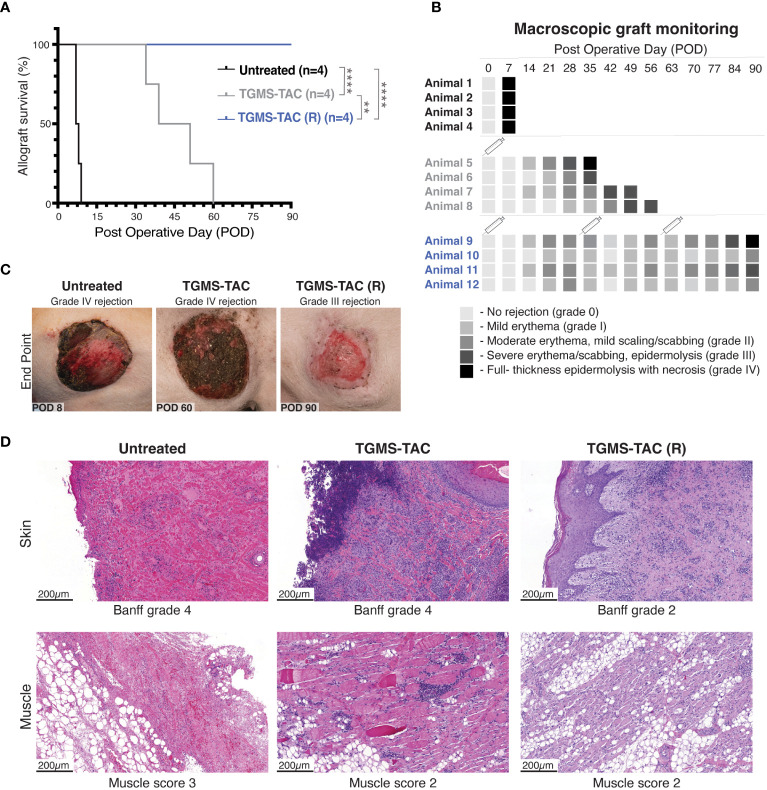
TGMS-TAC administration prolongs graft survival. **(A)** Kaplan-Meier survival curve of the three different groups (n = 4/group): untreated, TGMS-TAC single administration, and TGMS-TAC multiple administration (TGMS-TAC (R)). **p<0.01, ****p<0.0001 calculated by Log-rank (Mantel-Cox) test. **(B)** Macroscopic evaluation of graft survival during follow-up was graded as follows: 0 = no difference between graft skin and native skin, I = mild erythema, II = moderate erythema with the beginning of scaling and scabbing, III = severe erythema and scabbing with areas of epidermolysis, IV = full-thickness graft epidermolysis with areas of necrosis. Animals 5–8 were euthanized when reaching grade IV rejection at POD 35, 39, 51, and 60, respectively. A syringe indicates the time points of TGMS-TAC injection **(C)** Representative images of graft changes at endpoint (macroscopic grade IV rejection or POD90) with their corresponding grading. **(D)** Representative histological H&E staining of graft skin and muscle samples collected at endpoint. Skin was scored according to the Banff 2007 working classification. Muscle changes were graded as 0 = none, 1 = minimal, 2 = moderate, and 3 = extensive damage.

Having established the delivery system for TGMS-TAC, the multiple MHC-mismatched transplanted animals were either untreated (group 1), received a single dose of TGMS-TAC at POD 0 (group 2), or received multiple doses of TGMS-TAC (R) at 30-day intervals starting from POD 0 (group 3; **Figure 1B**). Recipient pigs were then followed until macroscopic grade III graft rejection or POD 90. As shown in **Figure 2A**, a single dose of TGMS-TAC (group 2, n=4) was sufficient to increase the median survival time (MST) to 45 days, compared to the untreated group (group 1, n = 4), which had an MST of 7 days. Monitoring of signs of rejection is depicted in **Figures 2B, C**.

Although treatment prolonged graft survival, Banff histopathological evaluation of skin graft samples collected at endpoint confirmed end-stage graft rejection for all pigs in both groups (**Figures 2B–D**). Graft muscle tissue isolated from pigs injected with TGMS-TAC was less damaged (**Figure 2D**, panel 2), showing muscle damage scores ranging from 0–2, compared to scores of 3 from muscles obtained from the untreated group. These muscle grafts from the untreated control group showed extensive damage, including necrosis and subtotal atrophy with moderate inflammation, whereas muscle samples from the TGMS-TAC treated groups only exhibited lipomatosis with mild to moderate perivascular infiltration of inflammatory cells.

Interestingly, all pigs treated with multiple TGMS-TAC injections (30-day intervals, TGMS-TAC (R) group, n = 4) reached the experimental endpoint of POD 90 (**Figure 2A**). However, while graft survival was prolonged, the grafts still showed macroscopic signs of rejection at the POD 90 endpoint (**Figures 2B, C**). Three grafts had Banff-skin scores of 2 showing moderate perivascular inflammation and mild epidermolysis, and one graft had extensive necrosis characteristic of Banff grade 4. As seen in **Figure 2D**, histologically, muscle-tissue samples of the TGMS-TAC (R) group had a similar profile: three of the grafts showed minimal perivascular-accentuated lymphohistiocytic infiltration, corresponding to a muscle rejection score of 2, and one exhibited extensive acute inflammatory infiltrate and necrosis with a muscle damage score of 3.

### 3.2 Local release of tacrolimus minimizes off-target toxicity while allowing high-intra graft TAC concentration

**Figure 3:**
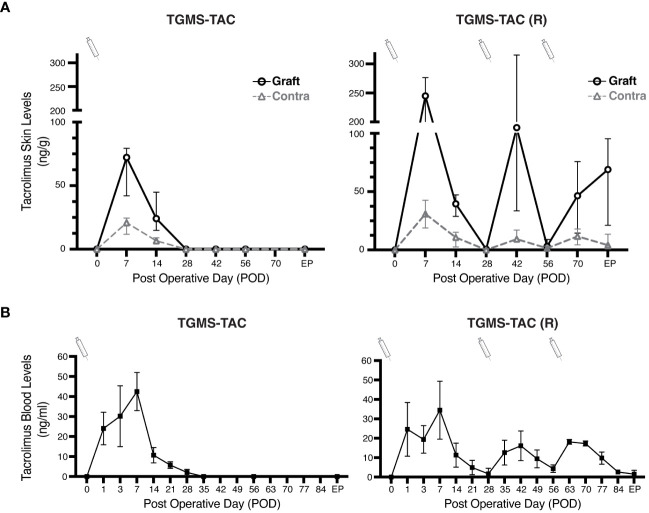
Local TGMS-TAC injections result in higher concentrations of the drug in grafts. **(A)** Comparison of TAC tissue levels in grafts and contralateral side skin for both single administration of TGMS-TAC (left), and multiple administration TGMS-TAC (R) (right). TAC levels were measured in skin biopsies collected on different postoperative days (POD). Tissue concentrations are represented as ng of TAC per g of skin. **(B)** Whole-blood levels of tacrolimus in pigs from the TGMS-TAC and (TGMS-TAC (R) groups were measured at POD 0, 1, 3, and then on a weekly basis. Blood levels are expressed as ng/ml. TGMS-TAC injection time points are indicated with a syringe. TAC was measured in both skin and whole blood using LC-MS/MS. Data are represented as means ± SD of n=4 samples.

Our hypothesis was that local TGMS-TAC administration would both allow a higher intra-graft concentration of TAC and avoid off-target toxicity due to its low systemic levels. We, therefore, assessed the distribution profile of TAC in both tissue and whole blood using Ultra-High Performance Liquid Chromatography Tandem Mass Spectrometry (LC-MS/MS). As illustrated in **Figure 3A**, the levels of tacrolimus in the skin of TGMS-TAC treated animals were notably elevated, ranging from 4- to 12-fold higher in the injected grafts compared to the levels detected in the contralateral skin. This substantial difference strongly suggests a localized release of tacrolimus at the site of injection, underscoring the effectiveness of TGMS-TAC to specifically deliver TAC to the desired area. Skin tissue levels of TAC measured over time revealed that the release peaked around POD 7 after the injection, corresponding to the time where rejection occurs in the untreated animals, further suggesting the inflammatory-based release profile. After POD 7, TAC levels gradually decreased, becoming undetectable by POD 28 (4 weeks after the initial injection). Pigs of the TGMS-TAC (R) group that received multiple injections of TGMS-TAC showed a similar profile (**Figure 3A**). A peak in TAC levels was observed 1 - 2 weeks after injection (POD 7, 37, 67) and then was non-detectable after 4 weeks (POD28 and POD56). These findings highlight that multiple TGMS-TAC injections are necessary to maintain appropriate drug levels in the VCA grafts.

We further evaluated the blood levels of TAC. As shown in **Figure 3B**, TAC blood levels corresponded to the profile found in graft skin tissue. Similar to what other DDS described (14, 21, 23), an initial burst release was observed within 7 days after the TGMS-TAC injection, which then progressively declined, reaching subtherapeutic levels 2 weeks after injection (<10 ng/mL). Unlike in the skin, blood TAC levels were higher than therapeutic target concentrations (>20ng/mL) from POD 1 to POD 7. This burst release of TAC was not observed with the subsequent TGMS-TAC administrations at POD 30 and POD 60 in the TGMS-TAC (R) group, where blood TAC levels were always below 20 ng/ml (**Figure 3B**). This finding suggests that the first systemic TAC peak was probably caused by the excess inflammation induced by the surgical procedure.

**Figure 4:**
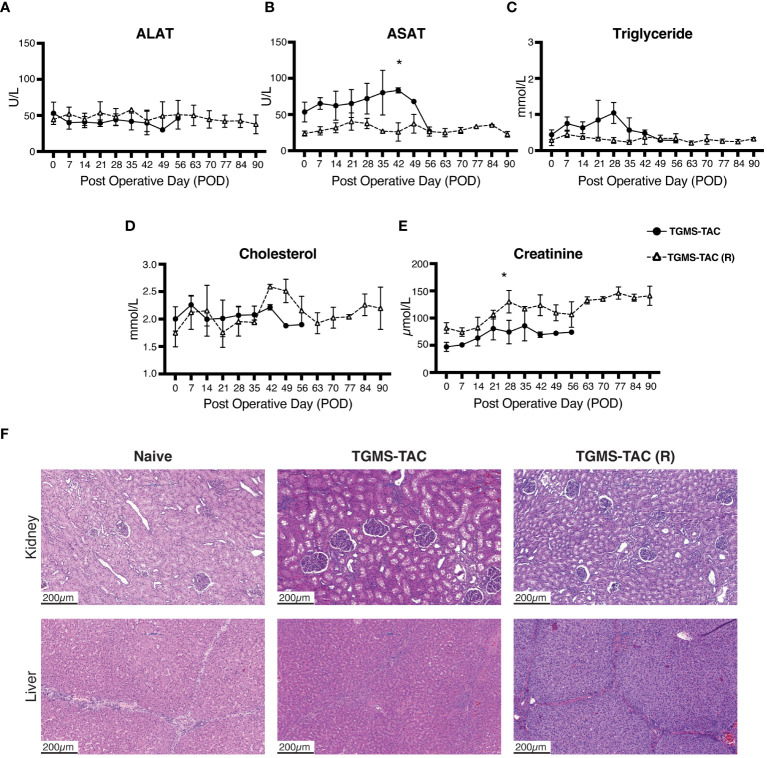
TGMS-TAC does not lead to off-target toxicity. Plasma levels of **(A)** alanine aminotransferase (ALAT), **(B)** aspartate aminotransferase (ASAT), **(C)** triglyceride, **(D)** cholesterol, and **(E)** creatinine as markers for liver and kidney off-target toxicity, respectively, during follow-up. Data are represented as means ± SD. *p<0.05 calculated by two-way ANOVA with Bonferroni correction for multiple comparisons. **(F)** Representative histological sections (H&E) of kidney and liver samples collected at endpoint from single-injection TGMS-TAC and multiple-injection TGMS-TAC (R) groups, respectively, compared with samples from a naive healthy pig.

To determine whether the transiently high concentration of TAC observed in the blood (>20 ng/mL) might cause off-target toxicity, plasma levels of ALAT, ASAT, triglyceride, cholesterol, and creatinine were measured (**Figures 4A-E**). While no effect on liver biomarkers was observed (**Figures 4A-D**), pigs of the TGMS-TAC (R) group, treated with multiple TGMS-TAC injections, showed minor elevations in creatinine (**Figure 4E**). However, these higher levels did not correspond to kidney damage. As shown in **Figure 4F**, histological evaluation of kidney sections revealed that glomeruli and tubules in cortical and medullary areas appeared normal compared to kidney biopsies from healthy naive pigs. Furthermore, no infiltration of inflammatory cells or signs of tubular damage were observed. As shown in **Figure 4F**, histological liver sections also confirmed the absence of hepatic injury. Lobular hepatic parenchyma and portal tracts were of regular appearance, and no signs of hepatocellular degeneration or damage to the biliary epithelium or sinusoidal dilation were detected.

### 3.3 Intra-graft TGMS-TAC administration dampens local immune response

**Figure 5:**
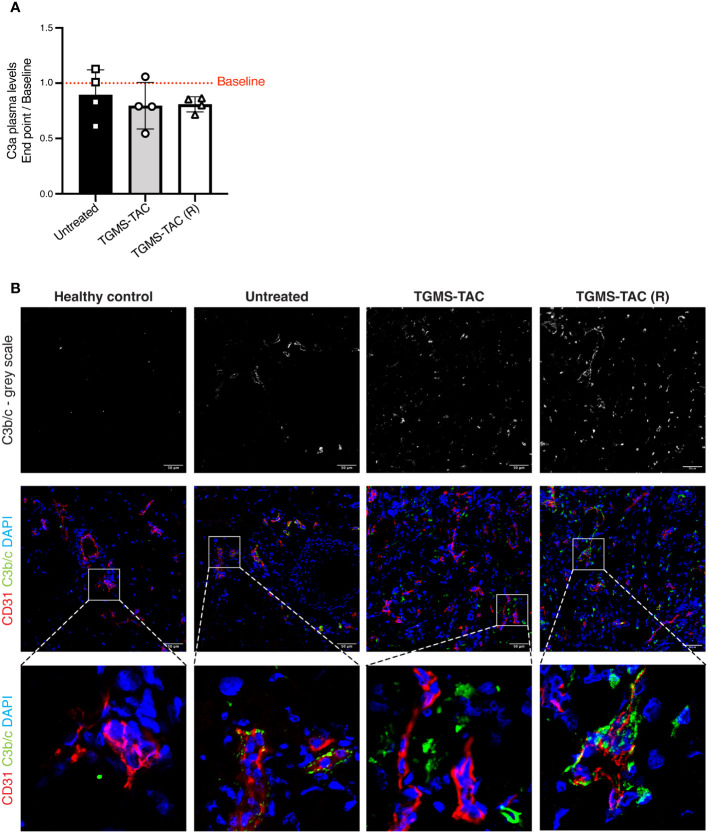
Complement activation is observed in rejected skin tissues but is not detected systemically. **(A)** Plasma levels of C3a quantified by ELISA. Data are represented as fold-changes at endpoint compared to baseline (endpoint/baseline). Data are individual values and means ± SD. Average baseline levels are denoted as a red dotted line. Statistical significance was assessed by one-way ANOVA with Tukey correction for multiple comparisons, p>0.05. **(B)** Representative immunofluorescence images of skin cryosections from grafts at endpoint compared to skin sections from a naive healthy pig. Skin was stained using DAPI for nuclei (blue), an anti-CD31 antibody as a marker for endothelial cells (red), and anti-C3b/c for complement deposition (green).

To better understand the role of intragraft tacrolimus administration in VCA, we evaluated T cells, neutrophils, complement, and inflammatory cytokines at endpoint both systemically in plasma samples, and locally in skin from grafts of treated and untreated animals. In VCA, skin is considered the most immunogenic component (51, 52), and was therefore chosen to analyze graft survival and/or rejection. Complement is known to participate in the immunological rejection of transplanted organs. Once activated, complement proteolytic fragments can directly mediate tissue injury and can also be important stimuli for tissue-resident immune cells (e.g., neutrophils and B cells) to produce chemokines and other inflammatory mediators that result in graft rejection (53). Since different activation pathways of the complement cascade merge at the complement protein C3, its C3a and C3b/c proteolytic fragments, were measured respectively in plasma and tissue. Surprisingly, no increase in C3a was observed in plasma samples at endpoint compared to baseline (**Figure 5A**). An average of 350 ng/mL of C3a was found in plasma samples from all groups. In contrast, C3b/c deposition was observed in all skin samples from rejected grafts (**Figure 5B**). Skin samples of all TAC-treated animals showed more prominent C3b/c staining when compared to grafts from untreated animals. It is known that tacrolimus activates complement during SOT (54), therefore, the increase of C3b/c deposition in rejected skin tissues of tacrolimus-treated animals is expected.

**Figure 6:**
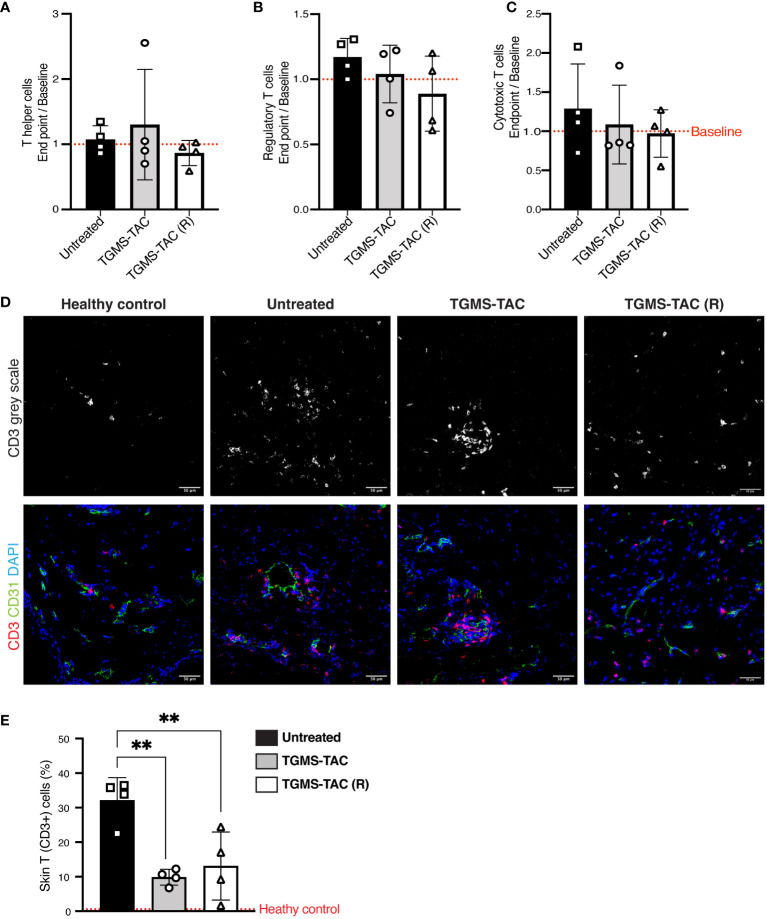
TGMS-TAC administration decreases local T-cell infiltration, with minimal systemic effects. **(A-C)** Flow cytometry identification of T-cell subsets from peripheral blood. Data are represented as fold changes at endpoint compared to baseline (composite baselines denoted as a red dotted line). T cells were identified as T helper (CD3+, CD4+, CD8-), Regulatory T cells (CD3+, CD4+, FoxP3+) and Cytotoxic T cells (CD3+, CD8+, CD4-). Data are shown as individual values with indication of mean ± SD. Statistical significance was assessed by one-way ANOVA with Tukey correction for multiple comparisons, p>0.05. **(D)** Representative immunofluorescence images of skin cryosections from grafts at endpoint compared to skin samples from a naive healthy pig. Skin was stained using DAPI for nuclei (blue), anti-CD31 for endothelial cells (green), and anti-CD3 for infiltrating T cells (red). **(E)** Quantification of T cells. The percentage of T cells in healthy naive skin is denoted as a red dotted line. Data are shown as individual values and mean ± SD. Statistical significance was assessed by one-way ANOVA with Tukey’s multiple-comparison test, **p<0.01.

Next, as T cells are one of the major players in graft rejection, subsets of circulating T-cells (i.e., T-helper, T-cytotoxic, and T-reg cells) were measured using flow cytometry. The gating strategies used for identifying the different T-cell subsets are shown in **Supplementary Figures S3A, B**. Interestingly, the frequency of the different T-cell subsets did not change at rejection compared to baseline. As shown in **Figures 6A–C**, TAC treatment did not alter the different T-cell subtypes. Similar amounts of T-helper (**Figure 6A**), T-cytotoxic (**Figure 6B**) and T-reg cells (**Figure 6C**) were found in all TAC-treated and untreated pigs. Among the subtypes, T-helper cells were found to be most abundant, with an average frequency of approximately 40%, followed by 15% T-cytotoxic cells and 2–4% T-reg cells in both TAC treated and untreated animals (**Supplementary Figures S3C-E**).

Since no difference in T-cell subsets was observed systemically, we explored whether local intra-graft TAC treatment would alter T-cell infiltration. A significant increase in T-cell infiltration (denoted as CD3+ cells) was seen in skin samples at rejection (**Figure 6D**). Interestingly, local treatment with TAC led to a 50% reduction of the amount of infiltrating T cells compared to untreated pigs. However, a 10% to 15% level of T-cell infiltration was still observed (**Figure 6E**) in the single- as well as multiple TGMS-TAC-treated pigs. This suggests that although intra-graft TGMS-TAC dampens local T-cell infiltration, it is not sufficient to completely prevent rejection by itself.

Next, we measured the plasma concentrations of various T-cell-derived cytokines (e.g., IFN-γ, IL-1β, IL-4, and TNF-α). Again, no differences in cytokine concentrations were identified at endpoint vs. baseline (**Supplementary Figure S4**), nor after treatment with TAC. Only the anti-inflammatory cytokine lL-1ra was found to increase in TGMS-TAC (R) treated pigs.

Taken together, these data indicate that although tacrolimus treatment delays rejection by dampening local T-cell infiltration, this is not sufficient to completely avoid rejection, suggesting that other cells might be involved.

### 3.4 Neutrophils and neutrophil extracellular traps are found in rejected grafts and are inhibited upon tacrolimus treatment

**Figure 7:**
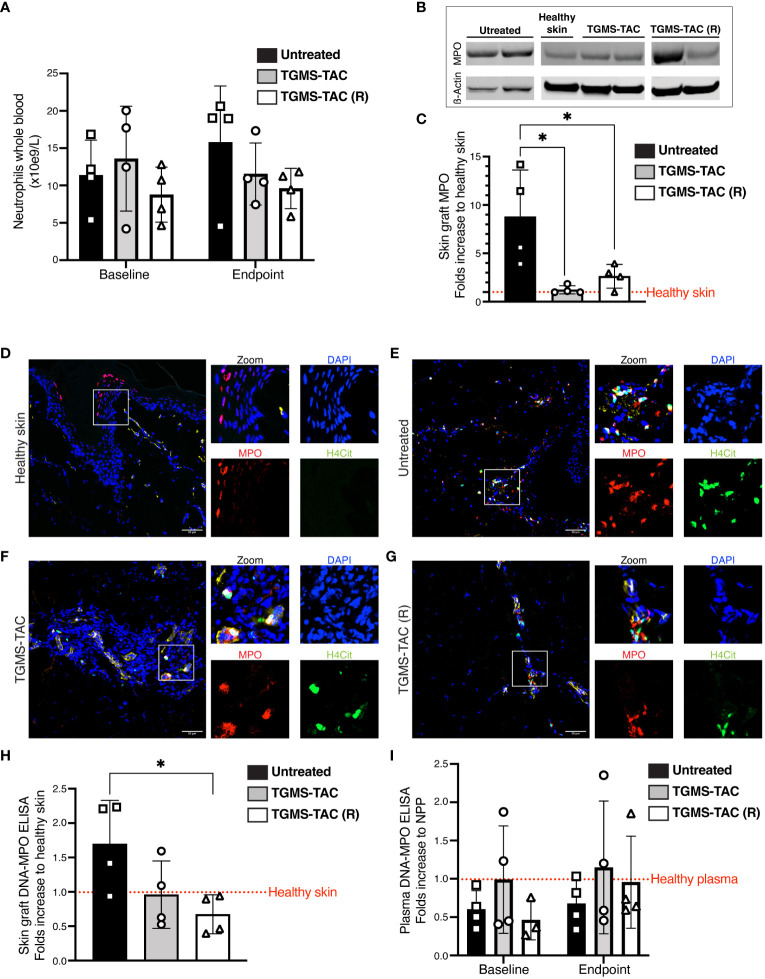
Graft-infiltrating neutrophils and NETs are found in skin tissue of rejected VCA grafts and are more prominent in untreated pigs. **(A)** Absolute frequency of circulating neutrophils measured from whole blood at baseline and at endpoint. Statistical significance was assessed by two-way ANOVA with Tukey correction for multiple comparisons, p>0.05. Data are shown as individual values and mean ± SD. **(B)** Representative western blot for myeloperoxidase (MPO) protein as a marker for neutrophil infiltration in graft skin. **(C)** Quantification of western blot data for MPO levels (ß actin control). *p<0.05 by two-way ANOVA with Tukey’s multiple-comparison test. **(D-G)** Representative immunofluorescence images of skin cryosections from grafts at endpoint compared to skin samples from a naive healthy pig. Skin was stained using DAPI for nuclei (blue), anti-MPO antibody as a marker for neutrophils (red), and anti-citrullinated Histone 4 (H4Cit) antibody (green) for NETs. **(H-I)** NET plasma levels in **(H)** graft skin and, **(I)** plasma, quantified by DNA-MPO ELISA. Statistical significance was assessed by two-way and one-way ANOVA, respectively. Data are shown as individual values and mean ± SD. **p<0.05. The dotted red lines represent composite baseline levels for naive healthy skin and plasma.

As neutrophils are also known to be involved in SOT rejection (36), we investigated their role in our VCA animal model. Similar to T cells, neutrophil levels in plasma did not increase at rejection (**Figure 7A**), nor did TGMS-TAC treatment influence neutrophil blood counts.

Since both complement activation and T-cell infiltration changes were mainly observed locally in rejected grafts, we analyzed neutrophil infiltration in rejected skin tissues. As shown in **Figures 7B, C**, neutrophils, identified as protein levels of MPO by western blot, were found in rejected skin grafts from all untreated animals. As observed in **Figure 7C**, MPO levels were significantly reduced in rejected graft skin lysates from animals treated with TAC.

During inflammation, neutrophils can release extracellular traps, a meshwork of decondensed DNA associated with neutrophilic proteins like MPO and citrullinated histones (37, 38). We, therefore, assessed the presence of NETs in skin of rejected grafts. NETs were visualized in all rejected grafts as DNA structures co-localizing with MPO and citrullinated Histone 4 (H4Cit). As shown in **Figures 7D–G**, NETs were less abundant in skin grafts after local treatment with TAC (**Figures 7F, G**) compared to grafts from untreated animals (**Figure 7E**). Next, NETs were quantified as DNA-MPO complexes by an in-house developed ELISA. (**Figure 7H**). A 40% to 60% decrease in NETs was detected in rejected skin tissue isolated from TAC-treated grafts compared to the untreated group. This indicates that TAC not only inhibited T-cell infiltration but also influenced neutrophil responses during rejection. NETs were also measured in plasma samples; however, in agreement with our previous findings that the events leading to graft rejection occur mainly locally, no NETs were detected (**Figure 7I**).

**Figure 8:**
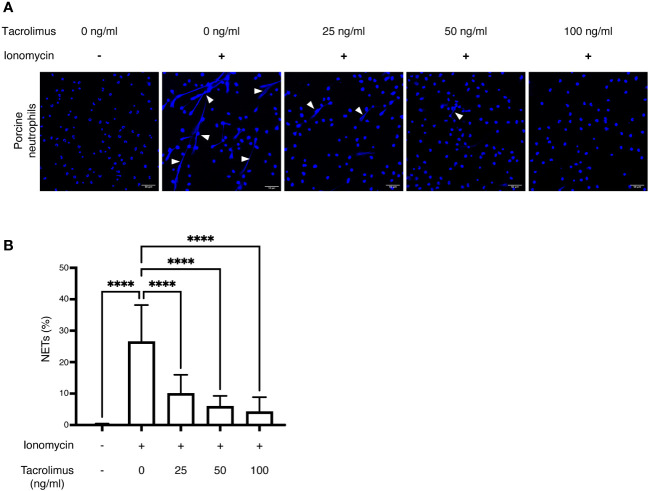
*In-vitro* inhibition of NET formation by tacrolimus: Isolated neutrophils from healthy pigs (n = 3) were stimulated using ionomycin to induce NET formation in the presence of different concentrations of TAC (0, 25, 50 and 100 ng/ml). **(A)** NETs were identified using DAPI (blue) and imaged through confocal microscopy. White arrowheads indicate extruded DNA content from neutrophils, confirming the presence of NETs. **(B)** NET percentages were calculated as the number of NETs/total number of neutrophils per field, using 5 representative images per condition. ****p<0.0001 by two-way ANOVA with Tukey’s multiple-comparison test.

As TAC appears to influence neutrophil responses during rejection, we also analyzed the effect of TAC on NET formation (NETosis) *in-vitro*. Porcine peripheral neutrophils were activated with ionomycin alone (a calcium salt known to induce NETosis) or after pre-incubation with different concentrations of TAC (**Figures 8A, B**). Preincubation of neutrophils with either 25, 50, or 100 ng/mL of TAC resulted in 62%, 77%, and 84% decreases in NETosis, respectively, when compared to ionomycin-stimulated neutrophils. This inhibitory effect of TAC on NET formation was further confirmed using human peripheral neutrophils (**Supplementary Figures S5A, B**). Taken together, these findings suggest that the presence of NETs may play an important role in VCA graft rejection and that their formation may be modulated by local immunosuppression.

## 4 Discussion

Currently, the main challenges for VCA are the frequent acute rejection episodes and the side effects of available immunosuppression therapies. Immunosuppression through drug delivery systems offers a beacon of hope in overcoming this hurdle by promising to prevent graft rejection with minimal off-side toxicity compared to standard systemic immunosuppression. While drug delivery systems loaded with immunosuppressive drugs have been proposed for various types of transplantation, such as heart (55), small bowel (56), and pancreatic islet cells (57), the intra-graft administration of immunosuppression using DDS holds particular promise in VCA. This is due to its specific anatomical composition and location, facilitating easier local and repetitive administration without requiring an extra surgical intervention i.e. intra-arterial administration in the pancreatic artery.

Here, using a clinically relevant VCA animal model, we show how graft survival can be prolonged while avoiding off-target toxicity utilizing TGMS-TAC, a locally administered on-demand drug delivery system. Our results show that multiple TGMS-TAC injections into the VCA graft skin result in prolonged graft survival, without increasing the risk of side effects. Although there was a mild increase in creatinine levels, no chronic damage in the kidney parenchyma was observed. The use of repeated, monthly TGMS-TAC injections allowed animals to reach the study endpoint of 90 days after transplantation. The graft survival observed in our study with a single injection of TGMS-TAC is similar to the one described by Fries et al, who used TGMS-TAC in a single MHC-mismatched pig limb transplantation model (14). However, for our study, we chose to use a clinically relevant model with respect to the number of MHC mismatches, thus being immunologically more challenging.

Compared to other drug delivery systems that offer only continuous drug release (20, 21, 23), hydrogels present a customizable delivery profile due to their adjustable physical properties, thus enabling drug release triggered by specific biological cues (58). Our previous research has demonstrated that TGMS-TAC utilizes enzyme-responsive bonds to facilitate drug release triggered by inflammation changes linked to graft rejection (12). This study reaffirms this capability, as no degradation of the hydrogel occurred upon TGMS-TAC administration in a setting lacking immunological challenges (no MHC mismatch). This presents the advantage of an on-demand mechanism, contrasting with alternative hydrogel systems such as ultrasound-sensitive (59) or those providing constant delivery (15).

Tissue and blood analysis of the TAC-treated and untreated animals showed no major differences in systemic levels of cytokines, circulating complement, T-cell subsets, neutrophils, NETs, and any other inflammatory markers at rejection when compared to baseline (before transplantation). This can partly explain why clinically in VCA, contrarily to solid organ transplantation, no biomarker has been identified as useful for non-invasive monitoring of rejection (60). Furthermore, in agreement with our findings, it has been shown that local mechanisms, rather than systemic ones, are key for determining the outcome of skin-containing VCA (61–63). This highlights the importance of achieving a high concentration of immunosuppressive medication in the graft area, where drug delivery systems such as TGMS-TAC offer an advantage over systemic administration. Indeed, local reinjection of TGMS-TAC led to increased tissue levels of tacrolimus over time, with subtherapeutic systemic levels, which was associated with sustained graft survival compared to single injection. Such minimal systemic levels of TAC avoid off-target kidney and liver toxicity and allow maintaining the circulating T cell levels essential to avoid opportunistic infection and lymphoproliferative disorders, which are well-known TAC-associated side effects. The beneficial impact of maintaining overall immune function through localized immunosuppression, such as TGMS-TAC, has similarly been observed in other drug delivery systems. Mayorga et al. (64) showed that following local administration of CTLA4Ig- and ATG-loaded DDS in a setting of pancreatic islet allotransplantation, circulating T regs levels remained stable over time, contrasting with a significant decrease observed in animals receiving systemic (intraperitoneal) administration.

For decades, T- and B-cells have been in the spotlight for immunosuppression therapies, as they have been considered to be the main mediators of rejection in VCA. Interestingly, in our study we found that not only T cells but also neutrophils are significantly increased in rejected VCA tissue, suggesting that innate immune cells may also play an important role in VCA rejection.

It has already been shown that neutrophil activation with subsequent NET formation can stimulate the innate immune response and impair graft survival in SOT (65). To our knowledge, the role of neutrophils and NETs has not been investigated in VCA so far. Here, we identified neutrophils and particularly NETs in rejected VCA grafts. In addition, we observed how local TAC treatment resulted in a significant decrease, but not complete inhibition, of infiltrating T cells and, surprisingly, also neutrophils and NETs. These findings suggest that local TAC treatment can decrease, in addition to T-cell proliferation, also NETosis in the skin.

Although multiple administrations of TGMS-TAC prolonged graft survival compared to untreated and single TGMS-TAC treated animals (MST of 90 days vs 7 and 45 days, respectively), all grafts showed signs of early rejection at the experimental endpoint. In our model, we suspect that once the TAC-loaded hydrogel is used up, and TAC tissue levels drop below a certain threshold, yet to be identified, both T cells, neutrophils, and NETs can be activated and invade the graft leading to rejection. In fact, the inhibitory effect of TAC on NETs was confirmed *in-vitro*. The required TAC concentration to significantly block NET formation is considerably high (>25 ng/mL), again suggesting that the local concentration of TAC needs to be maintained above the considered therapeutic level of 15 ng/mL. This tissue concentration is normally not achieved with systemic immunosuppression, wherein the best of cases reaches 5 ng/g (13, 66), or if achieved, it also results in significant kidney damage associated with supra-therapeutic levels. Achieving a local TAC tissue concentration of at least 15 ng/mL would require the use of a DDS. As an alternative to high-dose TAC treatment, other therapeutic interventions could be employed in order to dampen both adaptive and innate immunity. Blocking NETs has been suggested as a possible therapeutic intervention for both skin and lung allotransplants (65, 67). This suggests that a double therapeutic intervention using TGMS loaded not only with tacrolimus but also a NET-inhibitor could be a promising local therapeutic intervention for VCA.

It’s widely acknowledged that any mismatch in MHC carries a risk of decreased graft function and survival, with this risk escalating proportionally with the number of mismatched alleles (68). While achieving MHC matching in VCA isn’t feasible, a link has been identified between the frequency of acute rejection episodes and the number of HLA mismatches. Among MHCI and MHCII alleles, MHCI mismatches have shown a greater propensity to trigger rejection compared to MHCII mismatches (28, 30). This study utilized a stringent model incorporating multiple MHC mismatches, with MHCI alleles averaging at 85% and MHCII alleles at 67% mismatch, surpassing the reported average mismatch observed in human VCAs (29). Besides, our strategy involved exclusive monotherapy using TGMS-TAC, without any induction therapy. Remarkably, graft survival was significantly prolonged through intra-graft administration of TGMS-TAC. Moreover, research underscores the notable disparity in tacrolimus metabolism between pigs and humans, with pigs exhibiting considerably faster metabolic rates (69). This variance suggests that TGMS-TAC is likely to be depleted more rapidly in pigs compared to humans. Consequently, we hypothesize that in a clinical context, the application of TGMS-TAC may potentially extend graft survival even further.

This study has some limitations. First, it excluded a group undergoing systemic tacrolimus treatment, which is the current standard of care. The rationale behind this exclusion was based on the well-documented information regarding the acceptable graft survival time and associated off-target toxicity linked with systemic immunosuppression (9, 13). Second, since the study endpoint was 90 days, it would be necessary to assess whether prolonged exposure to TGMS-TAC results in a reduction of tacrolimus-related complications, particularly those that typically manifest later on, such as immunoproliferative and metabolic disorders. Extending the duration of the study to observe long-term effects is required as a next step to further strengthen the evidence supporting its clinical applicability. Second, an important aspect to consider is the difference in skin properties between pigs and humans. Although the fundamental aspects of the skin immune system remain comparable between the two species, which makes pig studies attractive as preclinical models, there is an increased thickness of pig skin compared to human skin (70). Given that TGMS-TAC is injected in subcutaneous depots throughout the entire skin surface, its application to larger areas like entire arms or cosmetically sensitive regions such as the face might result in prominent TGMS-TAC depots. This visibility may impact patient compliance and acceptance. Therefore, addressing these considerations and exploring strategies to mitigate them should be contemplated before its translation in clinical practice. Last, while our data implies the impact of local tacrolimus on NETs infiltration, further investigation is essential to elucidate the mechanism by which tacrolimus inhibits NET formation.

In summary, our findings demonstrate that repeated administrations of a tacrolimus-loaded hydrogel significantly prolong graft survival in a porcine VCA model. This is linked to increased levels of intra-graft tacrolimus and minimal off-target toxicity observed over a 90-day follow-up period, thus, underscoring the potential clinical relevance of drug delivery systems in VCA. Furthermore, our data uncover the presence of neutrophil extracellular traps (NETs) in VCA, shedding light on an aspect of the innate immune system that has been underexplored in the context of VCA rejection. This novel finding adds to the understanding of the effect of local tacrolimus immunosuppression, specifically on T cells and NETs.

## Data availability statement

The raw data supporting the conclusions of this article will be made available by the authors, without undue reservation.

## Ethics statement

The animal study was approved by the Veterinary Office of the Canton of Bern, Switzerland, approval number BE48/19. The study was conducted in accordance with the local legislation and institutional requirements. Experimental protocols were refined according to 3R principles. The studies involving humans were approved by Institutional Review Board of the University of Bern, Switzerland. The studies were conducted in accordance with the local legislation and institutional requirements. The participants provided their written informed consent to participate in this study.

## Author contributions

IA: Formal analysis, Investigation, Methodology, Project administration, Visualization, Writing – original draft, Writing – review & editing, Supervision. AH: Writing – review & editing, Investigation. AY: Investigation, Writing – review & editing. IL: Investigation, Writing – review & editing. SH: Investigation, Writing – review & editing. LZ: Investigation, Writing – review & editing. DC: Investigation, Writing – review & editing, Methodology, Supervision. LG: Investigation, Writing – review & editing. MP: Investigation, Writing – review & editing. SH: Investigation, Writing – review & editing, Formal analysis. TD: Investigation, Writing – review & editing. YB: Investigation, Writing – review & editing, Visualization. MM: Formal analysis, Investigation, Writing – review & editing, Visualization. MC: Writing – review & editing, Funding acquisition, Resources, Supervision. EV: Funding acquisition, Supervision, Writing – review & editing, Resources. GB: Writing – review & editing, Investigation. SA: Investigation, Writing – review & editing. JP: Investigation, Writing – review & editing, Formal analysis. AT: Conceptualization, Funding acquisition, Methodology, Writing – review & editing. PL: Resources, Supervision, Writing – review & editing. RR: Conceptualization, Funding acquisition, Project administration, Resources, Supervision, Writing – review & editing, Methodology, Validation. NS: Conceptualization, Formal analysis, Investigation, Methodology, Supervision, Visualization, Writing – review & editing, Validation. RO: Conceptualization, Funding acquisition, Methodology, Project administration, Supervision, Writing – review & editing, Investigation, Resources, Validation.

## Supplementary material

Supplementary Figure 1 MHC mismatches between donors and recipients. **(A)** Representative MHC typing between a donor and a recipient by low-resolution polymerase chain reaction. **(B)** Summary of mismatch percentages between donors and recipients in the different groups. p>0.05 by two-way ANOVA with Tukey’s multiple-comparison test.

Supplementary Figure 2 TGMS-TAC enzymatic release profile. **(A)** Graphical representation of the enzymatic release profile of TGMS-TAC, an intra-graft administered drug delivery system. **(B)** Cumulative release of TAC was assessed in-vitro over time. Lipase was added to the medium containing TGMS-TAC gel on day 28 as an enzymatic challenge. **(C)** Representative image of macroscopic graft changes at endpoint after syngeneic (left) and allogeneic (right) transplantation, with **(D)** ultrasound evaluation of TGMS-TAC presence in the grafts at endpoint. Red ovals depict the presence of subcutaneous TGMS-TAC depots in the grafts.

Supplementary Figure 3 Gating strategy for T-cell subsets from peripheral blood. Single cells were identified by their physical parameters (Forward-FSC and side-SSC scatter) and cell viability (viability-dye expression). CD3+ cells were gated and further identified as **(A)** T helper (CD3+, CD4+, CD8-), and Cytotoxic T cells (CD3+, CD8+, CD4-), and **(B)** Regulatory T cells (CD3+, CD4+, FoxP3+). **(C)** Flow cytometry identification of T-cell subsets from peripheral blood. Data are represented as the relative frequency of T-cell subsets out of total T cells (CD3+). T cells were identified as T helper (CD3+, CD4+, CD8-), Regulatory T cells (CD3+, CD4+, FoxP3+) and Cytotoxic T cells (CD3+, CD8+, CD4-). Data are shown as individual values, means ± SD. Statistical significance was assessed by two-way ANOVA with Bonferroni correction for multiple comparisons, p>0.05.

Supplementary Figure 4 Plasma cytokine levels. Cytokine concentrations (pg/mL) of **(A)** IL-1 alpha, **(B)** IL-1 ra, **(C)** IL-2, **(D)** IL–4, **(E)** IL-6, **(F)** IL-8, **(G)** IL-10, **(H)** IL-12, **(I)** IL-18, and **(J)** IFN gamma quantified by Luminex assay from plasma samples at baseline and at endpoint. Data are shown as individual values, mean ± SD. Statistical significance was assessed by two-way ANOVA with Bonferroni correction for multiple comparisons, *p<0.05.

Supplementary Figure 5 In-vitro inhibition of human NET formation by tacrolimus: Isolated neutrophils from healthy humans (n = 3) were stimulated using ionomycin to induce NET formation in the presence of different concentrations of TAC. **(A)** NETs were identified using DAPI (blue) and imaged using confocal microscopy. White arrowheads indicate extruded DNA content from neutrophils, confirming the presence of NETs. **(B)** NET percentages were calculated as the number of NETs/total number of neutrophils per field, using 5 representative images per condition. ****p<0.0001 by two-way ANOVA with Tukey’s multiple-comparison test.
