# Supplementary figures and images for: A local drug delivery system prolongs graft survival by dampening T cell infiltration and neutrophil extracellular trap formation in vascularized composite allografts
Source: Front Immunol. 2024 Jun 3;15:1387945. doi: 10.3389/fimmu.2024.1387945 (PMC11180892; doi:10.3389/fimmu.2024.1387945)

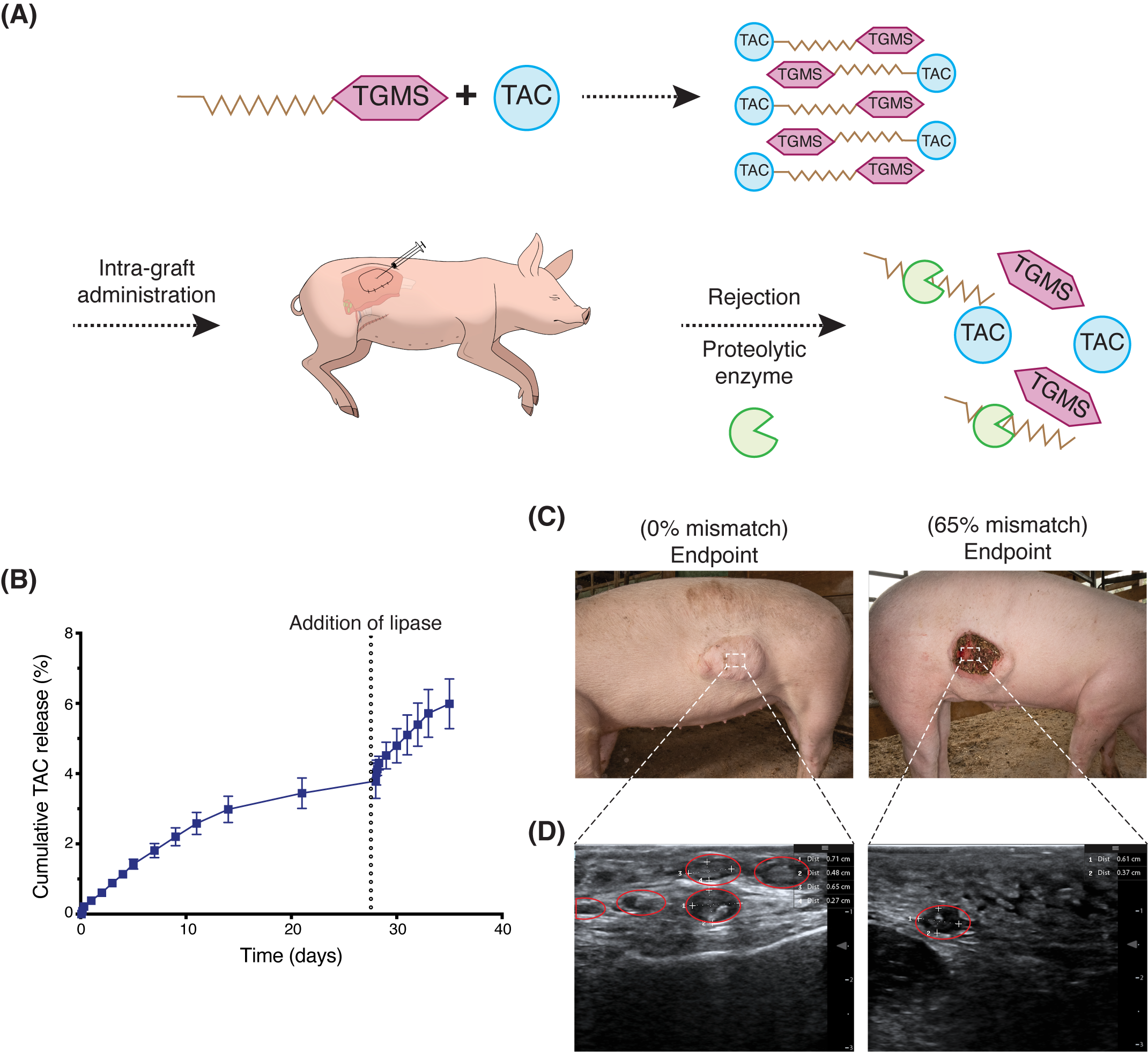

Supplement: Supplementary Figure 1 — MHC mismatches between donors and recipients. (A) Representative MHC typing between a donor and a recipient by low-resolution polymerase chain reaction. (B) Summary of mismatch percentages between donors and recipients in the different groups. p>0.05 by two-way ANOVA with Tukey’s multiple-comparison test. [file Image_1.tif]

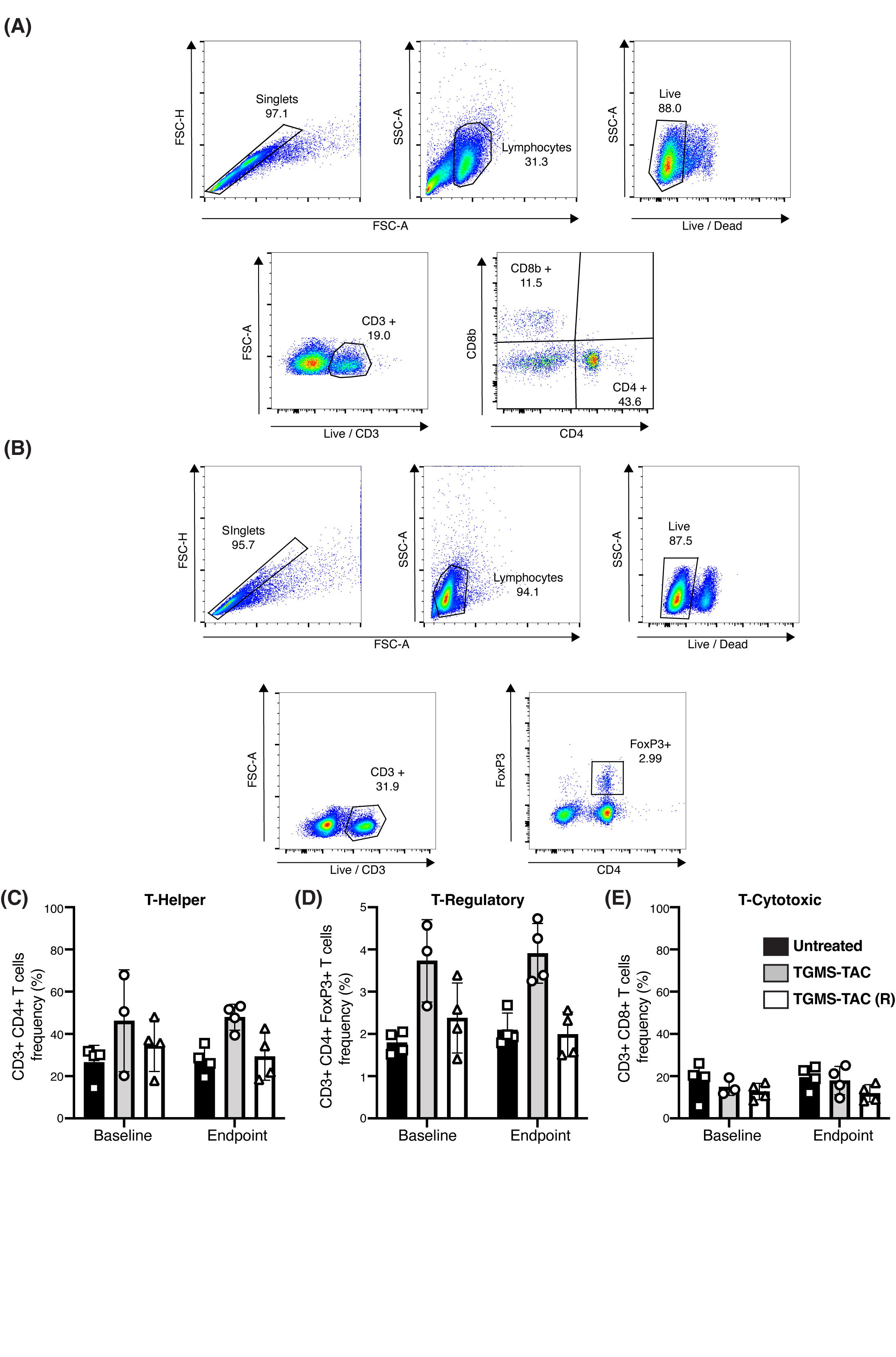

Supplement: Supplementary Figure 2 — TGMS-TAC enzymatic release profile. (A) Graphical representation of the enzymatic release profile of TGMS-TAC, an intra-graft administered drug delivery system. (B) Cumulative release of TAC was assessed in-vitro over time. Lipase was added to the medium containing TGMS-TAC gel on day 28 as an enzymatic challenge. (C) Representative image of macroscopic graft changes at endpoint after syngeneic (left) and allogeneic (right) transplantation, with (D) ultrasound evaluation of TGMS-TAC presence in the grafts at endpoint. Red ovals depict the presence of subcutaneous TGMS-TAC depots in the grafts. [file Image_2.tif]

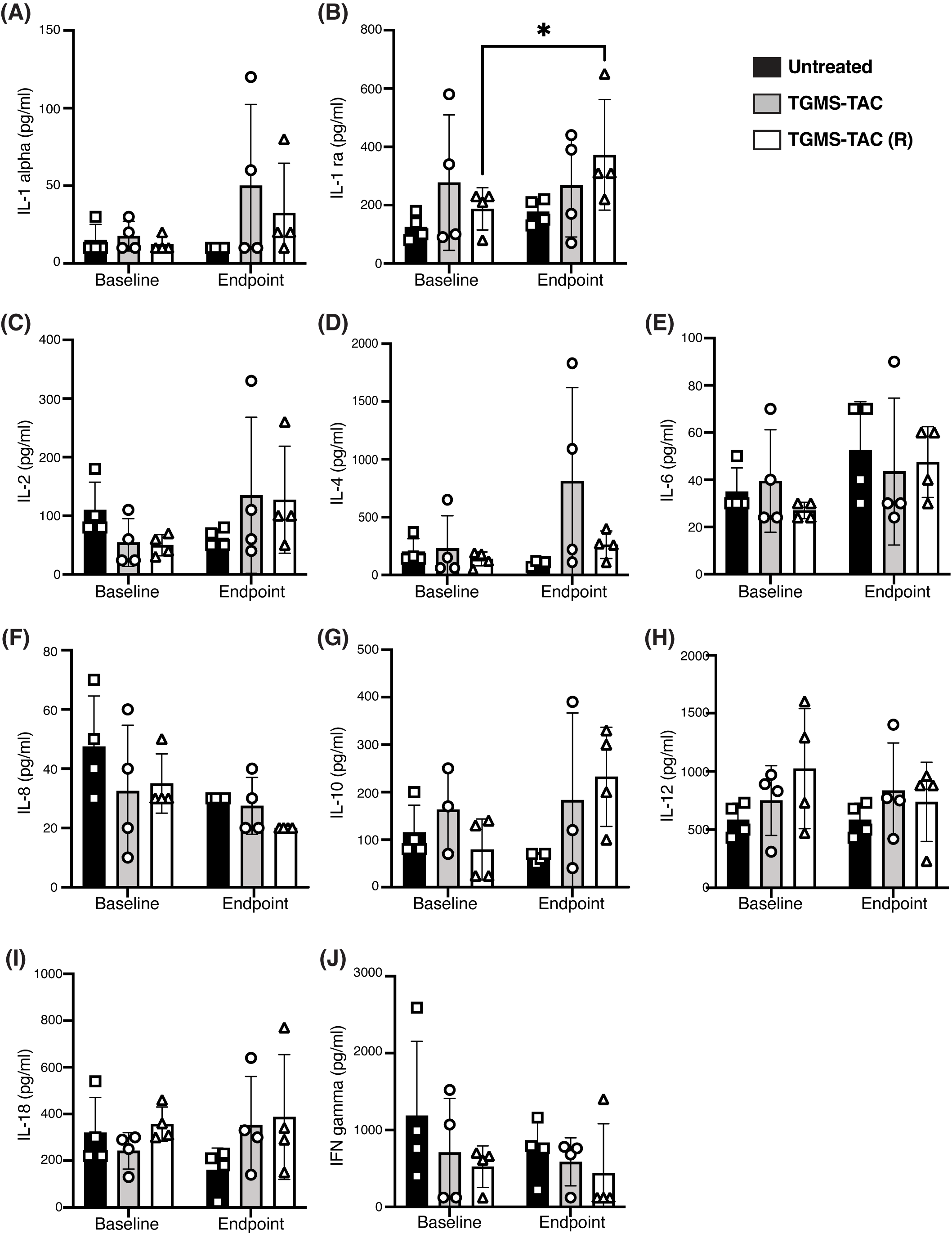

Supplement: Supplementary Figure 3 — Gating strategy for T-cell subsets from peripheral blood. Single cells were identified by their physical parameters (Forward-FSC and side-SSC scatter) and cell viability (viability-dye expression). CD3+ cells were gated and further identified as (A) T helper (CD3+, CD4+, CD8-), and Cytotoxic T cells (CD3+, CD8+, CD4-), and (B) Regulatory T cells (CD3+, CD4+, FoxP3+). (C) Flow cytometry identification of T-cell subsets from peripheral blood. Data are represented as the relative frequency of T-cell subsets out of total T cells (CD3+). T cells were identified as T helper (CD3+, CD4+, CD8-), Regulatory T cells (CD3+, CD4+, FoxP3+) and Cytotoxic T cells (CD3+, CD8+, CD4-). Data are shown as individual values, means ± SD. Statistical significance was assessed by two-way ANOVA with Bonferroni correction for multiple comparisons, p>0.05. [file Image_3.tif]

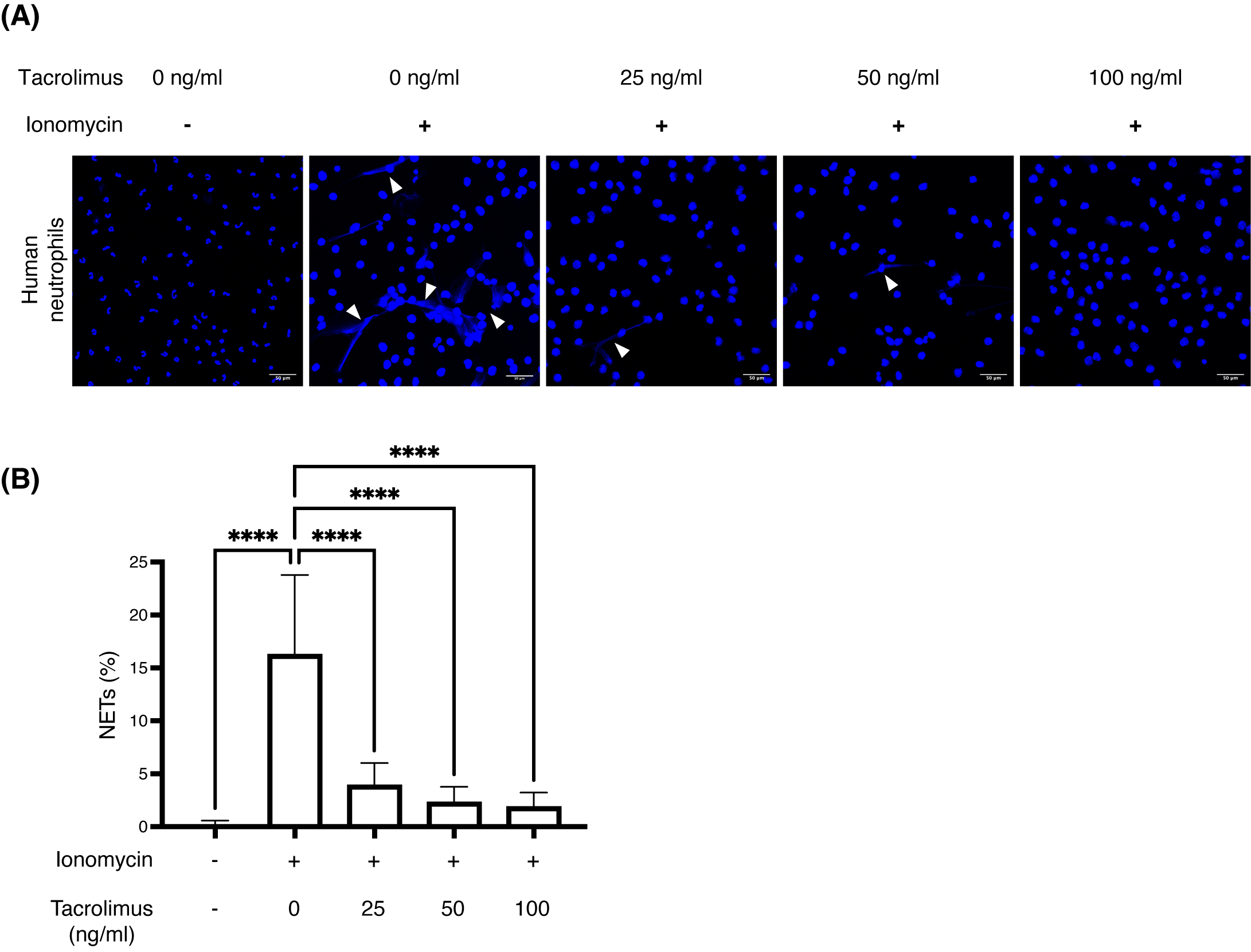

Supplement: Supplementary Figure 4 — Plasma cytokine levels. Cytokine concentrations (pg/mL) of (A) IL-1 alpha, (B) IL-1 ra, (C) IL-2, (D) IL–4, (E) IL-6, (F) IL-8, (G) IL-10, (H) IL-12, (I) IL-18, and (J) IFN gamma quantified by Luminex assay from plasma samples at baseline and at endpoint. Data are shown as individual values, mean ± SD. Statistical significance was assessed by two-way ANOVA with Bonferroni correction for multiple comparisons, *p<0.05. [file Image_4.tif]

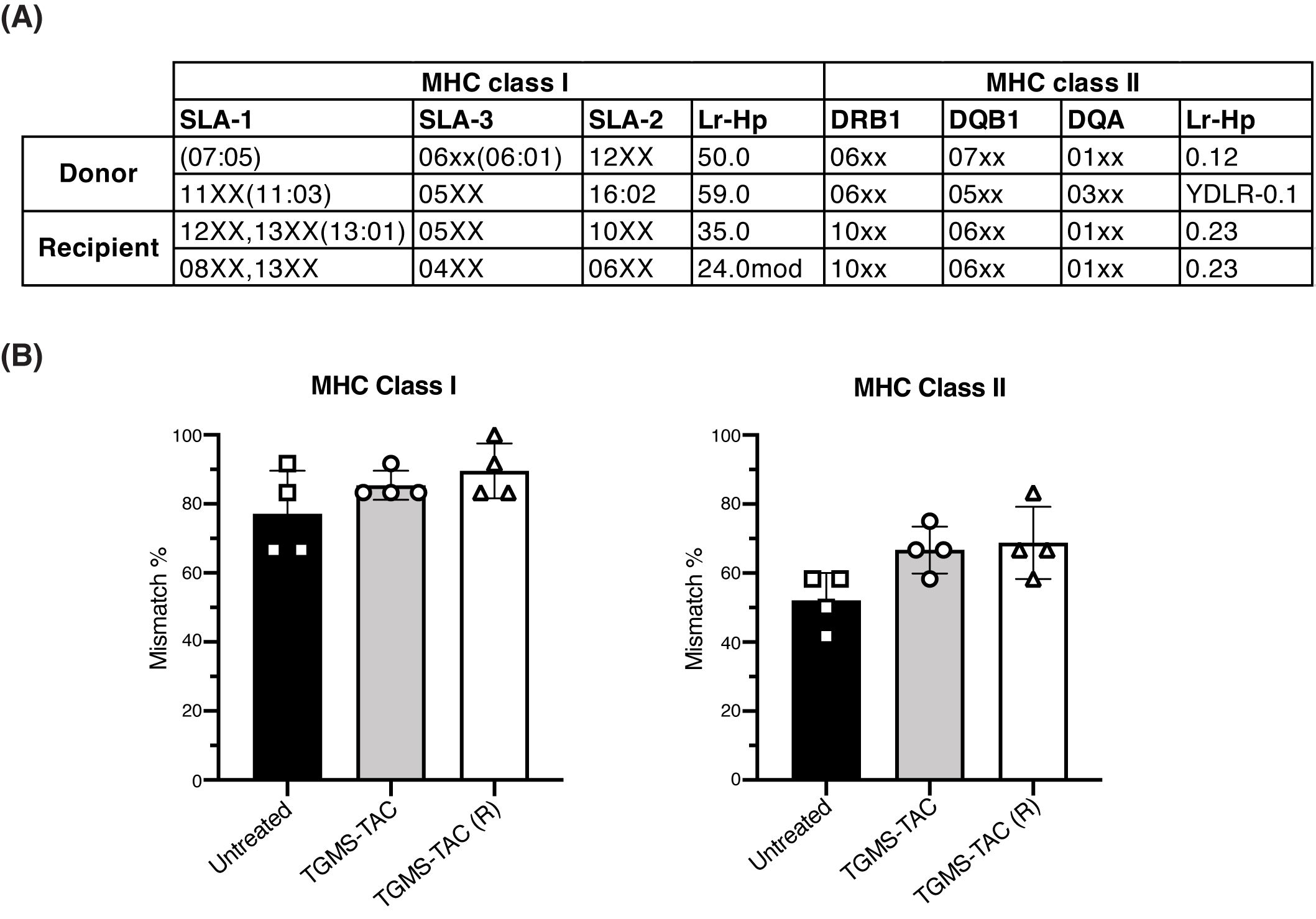

Supplement: Supplementary Figure 5 — In-vitro inhibition of human NET formation by tacrolimus: Isolated neutrophils from healthy humans (n = 3) were stimulated using ionomycin to induce NET formation in the presence of different concentrations of TAC. (A) NETs were identified using DAPI (blue) and imaged using confocal microscopy. White arrowheads indicate extruded DNA content from neutrophils, confirming the presence of NETs. (B) NET percentages were calculated as the number of NETs/total number of neutrophils per field, using 5 representative images per condition. ****p<0.0001 by two-way ANOVA with Tukey’s multiple-comparison test. [file Image_5.tif]
